# Is Self-Transcendence Philanthropic? Graded Response Model Approach

**DOI:** 10.3389/fpsyg.2022.816793

**Published:** 2022-05-18

**Authors:** Bandos Ros, Shinji Kaneko

**Affiliations:** ^1^Graduate School for International Development and Cooperation, Hiroshima University, Hiroshima, Japan; ^2^Department of Environmental Education, Ministry of Environment, Phnom Penh, Cambodia

**Keywords:** *altruism*, biosphere, volunteering, self-transcendence, Graded Response Model (GRM)

## Abstract

This study reveals that strong feelings of *altruism* were found to be statistically significant in explaining prosocial and pro-environmental behaviors. However, this was not the case for the latent trait *biosphere* in explaining pro-environmental behavior (e.g., past volunteering in clean-up activities). Regardless of whether they are overseas graduates or not, subjects in this study are more altruistic than biospheric by nature. Using the Graded Response Model (GRM) approach, the study found that the *biosphere* and *altruism* are obviously independent of each other and merging them into one dimension, in this instance referred to as “*self-transcendence*,” makes the construct less reliable. That is why this study in consistence with previous studies could not detect the effect of *self-transcendence* statistically, as it affects both the past volunteering in environmental affairs and the past volunteering in social welfare.

## Introduction

Values, the desirable goals and guiding principles in someone's life (see e.g., Brunsø et al., 2004; van der Werff et al., 2013), is a variable that is empirically studied in the field of environmental psychology to find its effects on behaviors. Some studies found that it exerts direct effects on behaviors (Stern et al., 1995; Liobikiene and Juknys, 2016), while others found its effect on behaviors via beliefs, attitudes, and norms (see Gärling et al., 2003; Nilsson et al., 2004; Lee, 2011; Maio, 2011; Steg and de Groot, 2012; van der Werff et al., 2013). Human values can drive a person's actions (Schwartz, 1992 as cited by Dominicis et al., 2017), particularly in inducing pro-environmental behavior (Crompton and Kasser, 2009; Knez, 2016). Though there is a value-action gap, Maio (2011) claimed that, in general, it influences a broad range of behaviors, for instance, waste recycling (Thomas and Sharp, 2013). In the field of environment, values are categorized into four different components (Stern et al., 1998; de Groot and Steg, 2008) (Steg et al., 2014): biospheric values (i.e., valuing the environment), altruistic values (i.e., valuing the welfare and well-being of other human beings), egoistic values (i.e., valuing personal resources/centrality), and hedonic values (i.e., valuing pleasure and comfort).

According to the theory of Values Beliefs Norms (VBN), values, especially biospheric values, determine environmental attitude (Stern et al., 1993, 1995; Hansla et al., 2008; Lee, 2011; Papagiannakis and Lioukas, 2012; van Riper and Kyle, 2014). The theory also confirmed that a person with altruistic values is more likely to act pro-environmentally (Stern, 2000 as cited by Prager, 2012). *Altruism*, defined as self-sacrifice with no apparent personal reward, was also found to be one of the drivers of volunteering (Unger, 1991; Okabe et al., 2017). Nevertheless, whether *altruism* is always associated with other types of volunteering or in different cultural contexts is doubtful/open to question. Though Becker (1974) provides evidence for an altruistic motive across many types of voluntary activity, Luria et al. (2017) suggested that people with different values in different cultures are likely to engage in different types of volunteering. Therefore, whether different volunteering fields are affected by various drivers should be explored further, as suggested by Kewes and Munsch (2019).

Altruistic values indicate one's concern for the harmful effects of the action on other human beings. So, altruistic values are more likely to be positively correlated with pro-environmental beliefs and behaviors when such behaviors result in positive outcomes for other people (e.g., Stern et al., 1998; de Groot and Steg, 2007; Perlaviciute and Steg, 2014). According to the Goal Framing Theory, environmental behaviors are governed by normative goals, where people are led to act based on what other people think they should do (Lindenberg and Steg, 2013; Steg et al., 2014). This means that altruistic people are likely convinced to engage in pro-environmental behaviors because they may think that protecting the environment is saving human lives. Therefore, Joireman et al. (2001) found that the more altruistic, the more concerned about the environment people will be, while Dominicis et al. (2017) confirmed that altruistic value was associated with pro-environmental behaviors.

Self-transcendence values, which make individuals focus on the interests of others and the environment (see e.g., Schwartz, 1992, 2012; Castelo et al., 2021; Wong et al., 2021), are led more by the normative goal (Steg et al., 2014a,b) and have been found to be positively related to pro-environmental beliefs and behaviors (Stern et al., 1998; Schultz and Zelezny, 1999; Boer and Fischer, 2013; Cheung et al., 2014; Steg et al., 2014b), such as energy saving, recycling, protection, and acceptance of environmental policies (Stern, 2000; Nordlund and Garvill, 2002, 2003; Milfont and Gouveia, 2006; de Groot and Steg, 2007, 2008, 2009; Hansla et al., 2008; Nilsson et al., 2016). Though it is well-known to predict volunteering behavior, *self-transcendence* was unexpectedly found to be statistically insignificant in explaining the variation in any form of volunteering (Onuki and Xiao, 2020). So, whether the *self-transcendence* value is reliable or not should be further investigated, as suggested by Stern et al. (1998). This study recalls Schwartz's value theory in which *altruism* and *biosphere* are combined into one value cluster, that is, the *self-transcendence* dimension (Dietz et al., 2005; Steg and de Groot, 2012). Though Stern et al. (1998) found the reliability of the construct, various empirical studies later found and supported the distinction between *altruistic* and *biospheric* value orientations (Schultz, 2000, 2001; de Groot and Steg, 2008; Steg et al., 2014b). In these contradictory implications, it is necessary to compare the effect of the one-factor construct, the *self-transcendence*, against the two-factor construct, *altruism* and *biosphere*, on different fields of volunteering.

## Materials and Methods

The study uses primary data collected from an online survey in November 2020. In cooperation with the Cambodian Association in Japan (CSAJ) and the Hiroshima Alumni Network and using social media (i.e., Facebook), a total of 224 respondents agreed and participated in the survey. The respondents are those scholars experiencing education locally (i.e., in Cambodia only) (*N* = 59), experiencing overseas education, but residing in their home country (i.e., Cambodia) (*N* = 65), and doing their education overseas (*N* = 100) (i.e., 85% in Japan).

Two indicators that are tested include past volunteering in blood or monetary donations and past volunteering in clean-up activities. These two variables are a binary indicator (1 = participated in clean-up activities, 0 = otherwise) and (1 = donated blood or money, 0 = otherwise). Participants were also asked to report their gender, age, educational level, and the fixed effects (i.e., location where they are residing: 1 = overseas education and living in their home country upon their graduation and 0 = otherwise and 1 = overseas education and still living in host countries and 0 = otherwise). These five background variables are included in our subsequent analyses as control variables.

For concerns on social and environmental issues, subjects were asked to rate the seven-point Likert scale on a nine-item measure (0 = extremely disagree to 6 = extremely agree). These items are adapted from a study measuring the validity and reliability of human values, such as *altruism, biosphere*, and *egoism* (Stern et al., 1998; Perlaviciute and Steg, 2014; Bouman et al., 2018). A row mean score or summative score of the three constructs is computed after the reliability and validity of the constructs are measured. Higher scores indicate people are more likely to exhibit *self-transcendent, altruistic*, and *biospheric* concerns (see Supplementary Table 1 for Descriptive Statistics). I computed the factor score of the three constructs as well, since their loadings were not similar. For instance, the loading of the one-factor model ranges from 0.58 to 0.90 (see Supplementary Table 3). The item that has a loading of 0.90 on a factor is more salient than that with a loading of 0.58. In this case, the factor score will be better than summative score to use because factor score weight items by their salience (Acock, 2013).

I used orthogonal factor analysis with varimax rotation to identify the internal structure of the items. In this analysis, nine items were loaded together. Based on its meaning and the Cronbach's alpha value (∞), item 6 was dropped, otherwise the internal reliability would decrease from ∞ = 0.89 to ∞ = 0.88. The item 7 value of Factor 1 and Factor 2 were close to each other, and it was then presumed to be an item of Factor 2 based on its meaning. I tested the Cronbach's alpha of Factor 2 with and without item 7, and the result showed exactly the importance of including item 7 in Factor 2, which makes the alpha value to increase from ∞ = 0.71 to ∞ = 0.79 (see Supplementary Table 2).

To test how the models will fit the data, I conducted confirmatory factor analysis (CFA), using the software package Stata 15.1. The CFA results show the one-factor model is a better fit with the data than the two-factor model. The model indices of the one-factor model are listed as follows: chi-square to the degree of freedom [$X_{\left(13\right)}^{2}$ = 20.232 with *p* > 0.05], Comparative Fit Index (CFI) is 0.991, root-mean-square error of approximation (RMSEA) is 0.05, and standardized root mean square residual (SRMR) is 0.030. The model indices of the two-factor model are listed as follows: chi-square to the degree of freedom [$X_{\left(16\right)}^{2}$ = 28.837 with *p* < 0.05], Comparative Fit Index (CFI) is 0.986, root-mean-square error of approximation (RMSEA) is 0.061, and standardized root mean square residual (SRMR) is 0.032 (see Supplementary Figure 1).

As already computed, the Cronbach's alphas of the one-factor model (∞ = 0.890) exceeded the criteria value of 0.7, indicating a high degree of internal consistency. Consistently, the internal reliability (rho) (*p* = 0.909) indicates that the variation in the scale is 90.9% (*p* = 0.909), which is explained by the construct (Acock, 2013). Similarly, the Cronbach's alphas of the two-factor model (i.e., biosphere and altruism construct) were ∞ = 0.893 and ∞ = 0.79 (>0.70), respectively, while the internal reliability (rho) was 87.7% (*p* = 0.877) and 94.5% (*p* = 0.945), respectively (see Supplementary Tables 3–5). From these results, it is presumed that the one-factor and two-factor constructs are reliable and valid (see e.g., Wong et al., 2021).

## Results and Discussion

The association between the two volunteering behaviors was first tested because altruism was found to be statistically correlated with the biosphere, as confirmed by Dominicis et al. (2017) and Joireman et al. (2001). Overall, 67.43% of respondents engaged in clean-up activities, of whom 72.25% engaged in blood or money donation. The odds of engaging in clean-up activities is 147/71 = 2.07. This means that there are 2.07 people engaging in clean-up for each person who has never engaged in such activity. The odds of engaging in clean-up activity, if subjects also engaged in blood or money donation, is 125/48 = 2.60. This means among those subjects donating blood or money, there are 2.60 people engaging in clean-up activity for each person who has never engaged in the clean-up. However, the odds of engaging in clean-up activity if subjects have never engaged in blood or money donation is 22/23 = 0.96. This means among those subjects who have never donated blood or money, there are just 0.96 people engaging in clean-up for each person who never participated in the clean-up activity. The odds ratio of engaging in clean-up is 2.60/0.96 = 2.71. So, the odds of engaging in clean-up if subjects donate blood or money are 2.71 times greater than the odds of engaging in clean-up if subjects never donate blood or money. How much greater? I found the difference to be 171% [100 × (2.71–1)] greater.

So, people engaged in social welfare are more likely to be engaged in clean-ups. To measure if the association is statistically significant, I employed chi-squared test, and the result was [$X_{\left(1,\text{N}=218\right)}^{2}$ = 8.877 and *p* < 0.01] with effect size φ (*phi*) = 0.202. It can be, therefore, concluded that there is a statistically significant association with moderate effect size between the two volunteering activities (see Table 1).

**Table 1:** Association between volunteering in clean-up and social welfare.

	**Response**	**Engaged in clean-up activity**		***P***	**Cramé's *V***
		**No**	**Yes**		
Donated blood or money	No	23 (51.11)	22 (48.89)	0.003^**^	0.202
	Yes	48 (27.75)	125 (72.25)		
	Total	71 (32.57)	147 (67.43)		

** *p < 0.01*.

To determine the predictors of these two past volunteer activities, a binomial logit model was applied. As hypothesized earlier, human values (i.e., *altruism, biosphere*, and *self-transcendence*) were used separately as the main predictor variables (or independent variables). The outcome variables (or dependent variables) are past volunteering in social welfare (i.e., blood or money donation) and past volunteering in clean-up activities. Each variable can be answered in the positive or negative, representing whether the respondents performed that activity. Multicollinearity between predictors was also tested using variance inflation factors (VIFs). The result showed that the mean VIFs for all the predictor variables is 1.39, which is acceptable.

Table 2 shows that self-transcendence values, which are hypothetically associated with both volunteering in social welfare and environment-oriented activities, were found to be statistically insignificant. A minor relationship was detected between the value and volunteering in the environment (regressions 15 and 16). However, no statistically significant relationship was found between the value and past volunteering in social welfare (as exhibited by blood or monetary donation). So, it could be concluded that self-transcendence value, which is expectedly associated with volunteering in both social and environmental services, is not supported by this study. This result appears to be inconsistent with previous ones (Stern et al., 1998; Boer and Fischer, 2013). However, it is consistent with a recent study, which reported that *self-transcendence* had no association with volunteering in any domain (Onuki and Xiao, 2020).

**Table 2:** Effects of human values on different fields of volunteering.

	**Volunteering in clean-up activities**			
	**Summative score**		**Factor score**	
	**[1]**	**[2]**	**[3]**	**[4]**
Biospheric values	0.426^+^	0.430^+^	0.264^+^	0.269^+^
	(0.219)	(0.226)	(0.145)	(0.150)
Marginal effect	0.093^+^	0.091^+^	0.057^+^	0.057^+^
	(0.047)	(0.047)	(0.031)	(0.031)
Controls	No	Yes	No	Yes
Observations (*N*)	218	210	217	209
	**Volunteering in blood or money donation**			
	**Summative score**		**Factor score**	
	**[5]**	**[6]**	**[7]**	**[8]**
Altruistic values	0.650^**^	0.675^**^	0.456^**^	0.467^**^
	(0.242)	(0.245)	(0.174)	(0.177)
Marginal effect	0.104^**^	0.110^**^	0.072^**^	0.075^**^
	(0.037)	(0.038)	(0.026)	(0.027)
Controls	No	Yes	No	Yes
Observations (*N*)	220	212	215	207
	**Volunteering in clean-up activities**			
	**[9]**	**[10]**	**[11]**	**[12]**
Altruistic values	0.305	0.298	0.189	0.195
	(0.206)	(0.213)	(0.147)	(0.151)
Marginal effect	0.067	0.063	0.041	0.042
	(0.045)	(0.045)	(0.032)	(0.032)
Controls	No	Yes	No	Yes
Observations (*N*)	218	210	213	205
	**Volunteering in clean-up activities**			
	**Summative score**		**Factor score**	
	**[13]**	**[14]**	**[15]**	**[16]**
Self-transcendence values	0.505^*^	0.512^*^	0.270^+^	0.280^+^
	(0.230)	(0.240)	(0.145)	(0.150)
Marginal effect	0.110^*^	0.101^*^	0.059^+^	0.059^+^
	(0.050)	(0.050)	(0.031)	(0.031)
Controls	No	Yes	No	Yes
Observations (*N*)	218	210	215	207
	**Volunteering in blood or money donation**			
	**[17]**	**[18]**	**[19]**	**[20]**
Self-transcendence values	0.278	0.274	0.189	0.186
	(0.273)	(0.278)	(0.173)	(0.177)
Marginal effect	0.046	0.046	0.030	0.030
	(0.045)	(0.046)	(0.028)	(0.029)
Controls	No	Yes	No	Yes
Observations (*N*)	220	212	217	209

*The vector of controls includes gender, age, education, and fixed effects. Standard errors are in parentheses*.

** *p < 0.01*,

* *p < 0.05*,

+ *p < 0.10*.

*Altruism*, being prosocial or self-less, was found to be statistically significant in predicting past volunteering in social welfare, with consistent results between summative score and factor score (regressions 6 and 8) as expected. From the marginal effect, we can conclude that with one unit increase in *altruism* (i.e., concerns for others), the probability of donating blood or offering money increases by 7.5%. So, the assumption that altruistic or prosocial behaviors positively relate to past volunteering in social welfare is accepted.

*Biosphere* was found to be statistically significant at 10% only (regressions 2 and 4). Thus, the assumption that biospheric value (i.e., concern for the environment) will positively correlate with past volunteering in the environment was unexpectedly rejected, in contrast with previous studies. Though a variety of behaviors are not always explained by human values (Maio, 2011), there is doubt and a question as to why *altruism* was found to be statistically significant, but the *biosphere* was not. Are subjects prosocial and pro-environment? To answer these questions, the strength of salience or sensitivity of each construct, “*altruism”* and “*biosphere,”* should be further explored. The question also applies to the construct, “*self-transcendence,”* since it was not statistically significant in predicting either of the two past volunteering behaviors.

**Figure 1:**
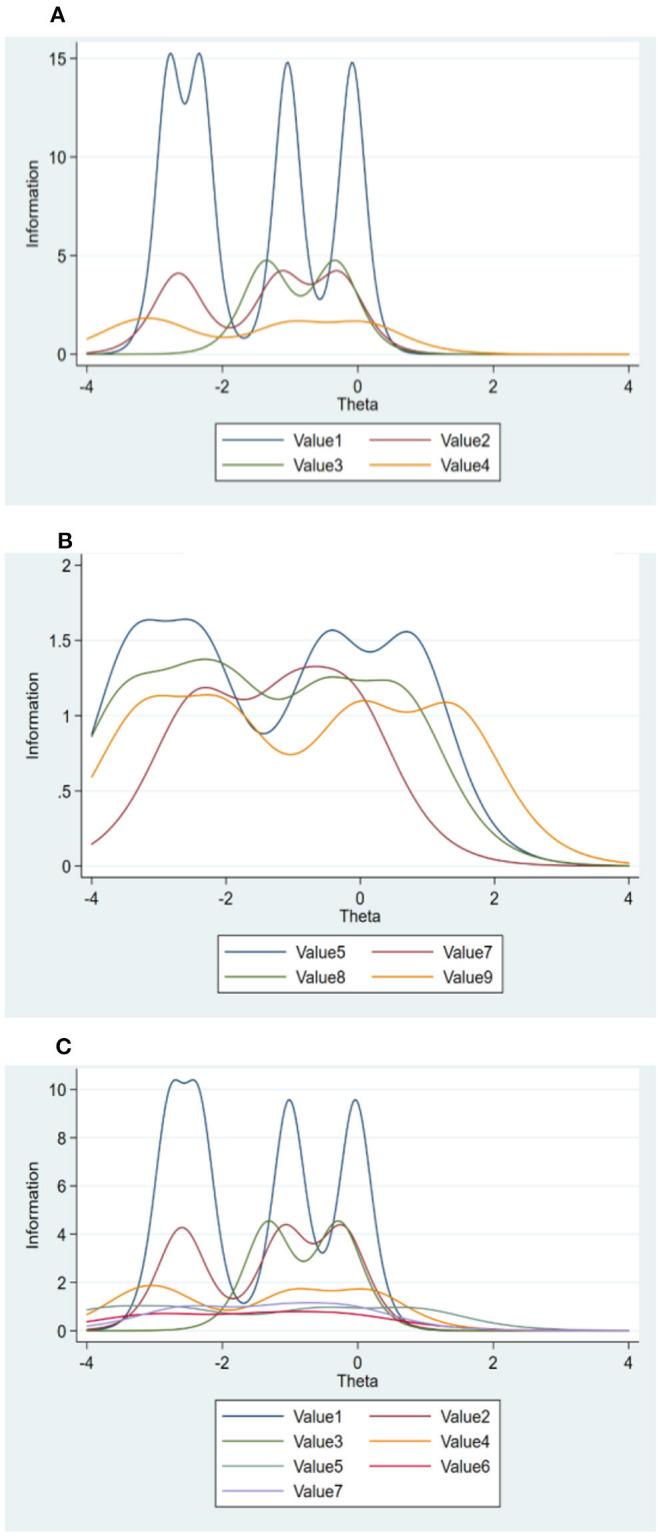
Strength of salience or sensitivity of human value (human concerns). **(A)** Item information function (Biosphere). **(B)** Item information function (Altruism). **(C)** Item information function (Self-transcendence).

To realize to what extent the subjects are altruistic or biospheric, or to detect the sensitivity of subject's *altruism, biosphere*, and *self-transcendence*, the Latent Trait Model (LTM), which is known as a scale assessment tool, was applied. Since the items to be measured are ordinal, the Graded Response Model (GRM) belonging to the item response theory (IRT) was utilized (Samejima, 1969). Based on the information provided by each item's response categories, an item information function (IIF) was developed for the three latent variables, that is, *biosphere*, a*ltruism*, and *self-transcendence*. Figure 1 shows the graphs for each item's information function. The graphs were created using the Stata 15.1 package (Acock, 2018).

As seen in Figure 1, the peaks in each graph represent the location on which the item of underlying value latent trait (i.e., human concerns) provides information the most. Because the Information is the reciprocal of the standard error of measurement (i.e., the variance of the latent trait level) at a given point on the latent trait, the more information, therefore, means the less error. The amount of information basically indicates an item's ability to measure the value latent trait reliably (Bankert et al., 2017). For reliability of the item's ability, please see Table 3.

**Table 3:** Reliability for the human values of θ (*theta*) from the GRM model.

**θ**	**Reliability**		
	**Biosphere**	**Altruism**	**Self-transcendence**
−4	0.459	0.712	0.684
−3	0.925	0.829	0.927
−2	0.882	0.829	0.902
−1	0.959	0.812	0.956
0	0.955	0.831	0.954
1	0.408	0.789	0.700
2	-	0.557	0.330
3	-	0.172	0.075
4	-	0.026	-

The strength of human concern lies on the x-axis and has, in its transformed scale of *theta*, a mean of 0 and a standard deviation of 1 with a somewhat arbitrary range that covers the extent of the latent trait (Bankert et al., 2017). For the sake of this research, the *theta* for human concern strength ranges from −4 to 4, with those closer to −4 displaying lower levels of concern and those closer to 4 displaying higher levels of concern. Thus, an information function peaking closer to 4 provides a considerable amount of information at high levels of concern, whereas an information function that peaks closer to −4 better captures lower levels of concern. Finally, an information function that peaks at the midpoint presents an item with considerable ability to distinguish middle from higher and lower levels of concern. Thus, ideal items should cover a wide range of subject's concern strengths and be able to discriminate effectively among different levels.

From the information provided in the latent trait, *self-transcendence*, the seven items do not supplement each other, which is at odds with the other two latent traits, the *biosphere* and the *altruism*. The items belonging to *biosphere* (i.e., *value 1, value 2, value 3*, and *value 4*) are up above, while the items belonging to *altruism* (i.e., *value 5, value 6*, and *value 7*) are far below (Graph A, Figure 1). In this case, the three items belonging to *altruism* provide the least information. Combining these two constructs into one *self-transcendence* dimension more likely provides more information on the *biosphere* than the *altruism* side. The primary conclusion is, therefore, that *altruism* and *biosphere* are more likely two separate constructs (Schultz, 2000, 2001; de Groot and Steg, 2008; Steg et al., 2014b) which should not be merged into *self-transcendence* dimension, as reported in previous studies of Stern et al. (1998) and Boer and Fischer (2013).

On the contrary, regarding the information provided in the latent trait, *altruism* and *biosphere*, the items of each construct supplement each other to cover a broad range of the latent trait of human concern on social and environmental issues. For the *biosphere*, value 1: “*It is important to love nature more*” provides the most information, while value 4: “*It is important we shall live with nature”* provides the least information. Value 2: “*It is important to stop environmental pollution*” supplements with value 3: “*It is important to protect and preserve environment*,” where there is low information provided by value 2 (i.e., θ = −2) and is covered by the information provided by value 3 (Graph A, Figure 1).

In Graph B (Figure 1), the four values supplement each other well. Value 5: “*It is important to help each other*,” value 8: “*It is important we shall have equal opportunity*,” and value 9: “*It is important we should take care of those who are worse off* ” display multiple peaks above the midpoint of the latent trait continuum and thus provide good coverage of higher level of concerns for other people. Value 7: “*It is important to have equal justice*” provides more information below the midpoint of the latent trait though. So, if the subjects of this study are less altruistic, they believe that equality-based justice is important, but if their concerns are high (i.e., θ > 0), they more likely agree that the other three values (i.e., helping people, providing equal opportunity, and taking care of those who are worse off) are more crucial.

The Reliability of the fitted IRT model can be computed with the following equation:


(1) $$\begin{array}{r} \rho_{i}=1-\sigma_{e}^{2} \end{array}$$

The error variance $\sigma_{e}^{2}$ is defined as 1/*I*, where *I* is the information. At some points on the scale, there is very little error variance, while at the other points, there is a lot. The error variance is small when there is a lot of information and larger when there is less information (Thissen, 2000). So, Equation (1) could be written again as:


(2) $$\begin{array}{r} \rho_{i}=1-\sigma_{e}^{2}=1-\left(1/I_{i}\right) \end{array}$$

This formula defines the reliability of the measure at different points, *i*, along the θ continuum (Acock, 2018).

As mentioned earlier, the items representing *value 1* of the latent trait *biosphere* provide the most information, when compared to the other items (Graph A). The reliability *P*_−3_ = 0.925 means only 0.075 (7.5%) of the variance in the measure of the people who have low *biospheric* levels θ = −3 is not reliable (Table 3). Though multiple peaks were detected among the four items, the reliability of the information provided is high only when the θ value is between −3 and 0, so it is more likely that the subjects of this study are less *biospheric* (i.e., less concern for environment or the subjects are neither pro-environmentalist nor environmental activists). If the subjects of this study have stronger concern (i.e., θ > 0), the influence of biosphere on past volunteering in clean-up would have likely been statistically detected. It could be presumed that the subjects of this study strongly value nature and environment (*M* = 5.36, *SD* = 0.66, α = 0.893), but do not see themselves as people who act pro-environmentally (Lorenzoni et al., 2007; Gössling et al., 2009; see, e.g., van der Werff et al., 2013; Juvan and Dolnicar, 2014). This gap could occur because people do not acknowledge environmental problems or because they do not believe that these problems could or should be mitigated via individual actions and thus deny or displace individual responsibility.

In contrast, the items of latent trait *altruism* supplement each other (Graph B). The reliability *P*_1_ = 0.789 means 78.9% of the variance in the measure of the people who have a high *altruistic* level (θ = 1) is reliable (Table 3). It is concluded that the subjects of this study are more altruistic (i.e., more concerned for others, prosocial, or selfless).

### Why *Altruism* Does Not Have Association With Past Volunteering in Clean-Up Activities?

**Figure 2:**
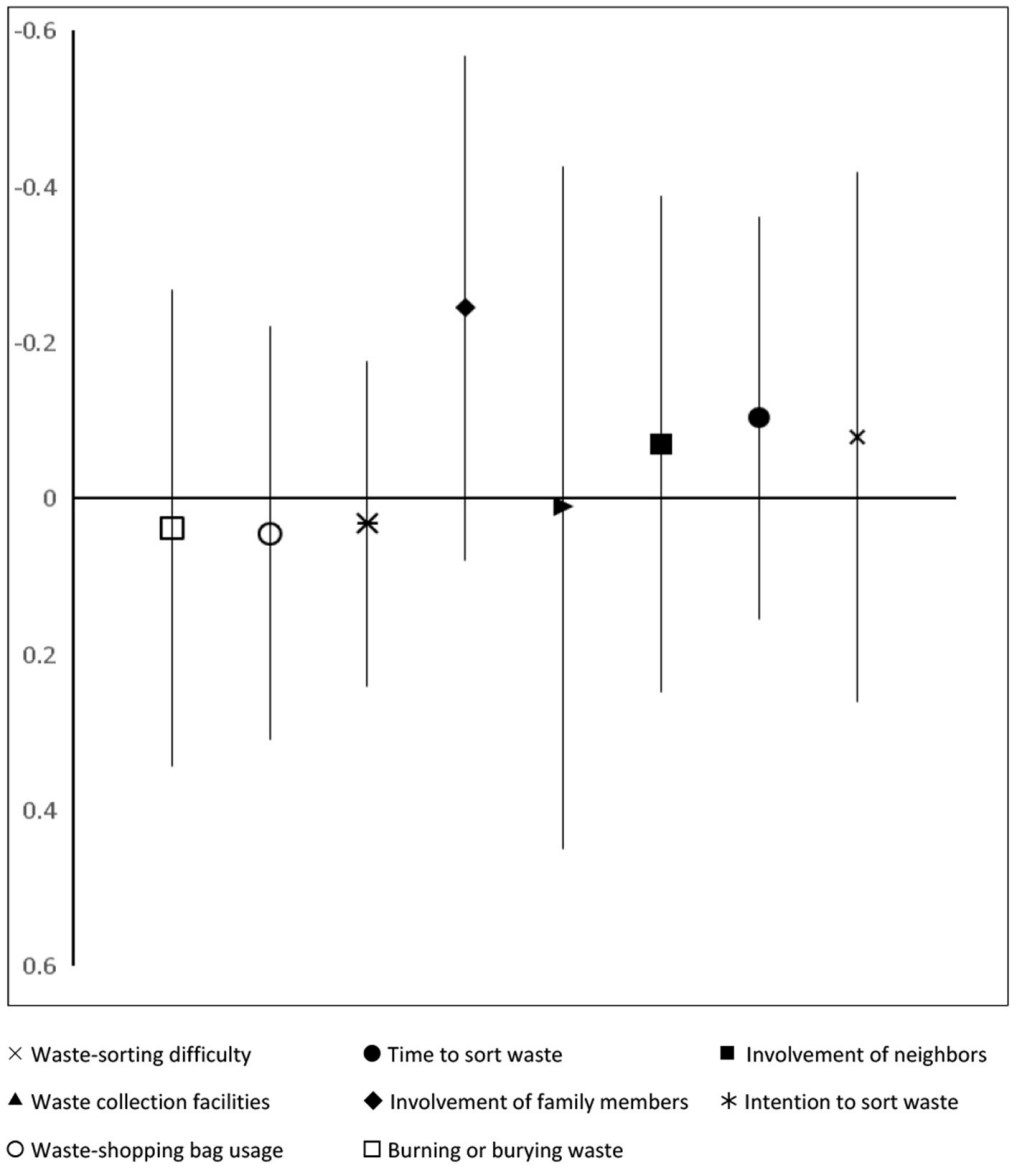
Effects of past volunteering in clean-up on pro-environmental knowledge, intention, and practices. ×, Waste-sorting difficulty; •, Time to sort waste; ■, Involvement of neighbors; ▴, Waste collection facilities; ■, Involvement of family members; *, Intention to sort waste; °, Waste-shopping bag usage; □, Burning or burying waste. The plot shows the statistically insignificant effects of volunteering in clean-up activities on environmental knowledge and pro-environmental practices. Full regression results are reported in Supplementary Table 6.

As confirmed by GRM, the construct “*altruism”* was found to be reliable, and it should have, therefore, been found to be statistically significant in predicting the past volunteering in clean-up (Joireman et al., 2001). But, this is not the case (regressions 10 and 12, Table 2). The reason behind this is that whether people volunteer or not is also related to obligation (as payback, social/parental pressures, and need to conform/win approval) (Wiehe and Isenhour, 1977 as cited by Henderson, 1981; Unger, 1991). If this is true, the association between the past volunteering in clean-up and knowledge, attitudes, and behavior toward the environment may be statistically insignificant. Using OLS, I regressed the effect of past volunteering on eight items assessing the knowledge, attitudes, and behavior. Figure 2 clearly shows that there is no statistically significant difference between those subjects who had volunteered or had not volunteered in terms of their environmental understanding, as well as their pro-environment intention and practices. So, this finding is consistent with the past studies, which reported that obligation (i.e., something needs to be done) drives people to volunteer (Wiehe and Isenhour, 1977 as cited by Henderson, 1981; Unger, 1991).

## Conclusions

The latent trait *self-transcendence* (i.e., combining *altruism* and *biosphere*) is not found to be reliable and valid until two items (i.e., item 8: *It is important we shall have equal opportunity* and item 9: *It is important we should take care of those who are worse off*) are dropped from the model. In this way, its effect on prosocial and pro-environmental volunteering was found to be statistically insignificant, which more likely resulted from the independence of *altruism* from *biosphere* (Table 3). Though the high correlation between the two constructs was statistically detected, merging *altruistic and biospheric values* into *self-transcendence* dimension appeared to be vague. This result more likely reflects the reasons behind the study of Onuki and Xiao (2020), who found that *self-transcendence* had no association with volunteering in any domain. At the same time, this result also explains why the latent trait *biospheric* value (i.e., concerns for environment) did not affect the past volunteering in the environment, while the latent trait *altruistic* value did affect the past volunteering in social welfare. It is, indeed, because the subjects in this study have a lower level of concern for the environment than others.

## Author's Note

I am interested in behavior change in the context of environment. Since my background is more related to environmental education, I look at values-related factors to understand its effect on human behavior change. There are many studies on human values and it seems various findings were found by the same factors such as self-transcendence. With the new method of GRM, we could explore in more detail the underlying reasons behind those findings. In short, my study found the construct 'self-transcendence' which is the combined altruistic and biospheric concern is invalid or less reliable since the two constructs are obviously independent from each other. Pro-environmentalist is more likely different from pro-socialist. However, I suggest to look at more deeply on this issue with bigger population or even in different cultural contexts.

## Data Availability Statement

The data used to support the above findings is available from the corresponding author upon a reasonable request.

## Ethics Statement

Ethical review and approval was not required for the study on human participants in accordance with the local legislation and institutional requirements. Written informed consent for participation was not required for this study in accordance with the national legislation and the institutional requirements.

## Author Contributions

All authors listed have made a substantial, direct, and intellectual contribution to the work and approved it for publication.

## Conflict of Interest

The authors declare that the research was conducted in the absence of any commercial or financial relationships that could be construed as a potential conflict of interest.

## Publisher's Note

All claims expressed in this article are solely those of the authors and do not necessarily represent those of their affiliated organizations, or those of the publisher, the editors and the reviewers. Any product that may be evaluated in this article, or claim that may be made by its manufacturer, is not guaranteed or endorsed by the publisher.
