# Supplementary material for: Is Self-Transcendence Philanthropic? Graded Response Model Approach
Source: Front Psychol. 2022 May 18;13:816793. doi: 10.3389/fpsyg.2022.816793 (PMC9159297; doi:10.3389/fpsyg.2022.816793)

Supplemental Information (SI)

Is Self-Transcendence Philanthropic? Graded Response Model (GRM) Approach

TABLE 1 | Descriptive statistics.

Variables	Mean or share	SD	Min	Max
How often you use shopping bag	2.64	0.99	0	4
Intention to sort your garbage	3.36	0.72	0	4
How often you sort your garbage	2.64	1.25	0	4
I agree that my garbage is difficult to separate	1.74	1.12	0	4
I agree I do not have time to separate my garbage	1.23	0.88	0	4
I agree waste-sorting needs the involvement of my neighbors	2.43	1.09	0	4
I agree I won't sort my garbage till waste-sorting facilities are installed	2.29	1.40	0	4
I agree waste-sorting needs the involvement of my family members	2.13	1.03	0	4
I agree my garbage should be burned or buried	2.44	0.95	0	4
Self-transcendence value (p = 0.909) [summative score]	5.27	0.62	3.28	6
Biospheric value (p = 0.877) [summative score]	5.36	0.66	3	6
Altruistic value (p = 0.945) [summative score]	4.90	0.70	2.25	6
Age: years old	30.97	6.37	18	52
Education level: Numbers of year in education	17.42	1.60	12	22
Male: Dummy (1 = male)	0.59	0.49	0	1
Overseas education (home-country): Dummy (1 = yes)	0.45	0.50	0	1
Overseas education (host-country): Dummy (1 = yes)	0.29	0.45	0	1
Volunteering in clean-up: Dummy (1 = yes)	0.67	0.47	0	1
Volunteering in blood or money donation: Dummy (1 = yes)	0.79	0.41	0	1

TABLE 2 | Factor Loading after Varimax Rotation.

	Two-Factor		One-Factor
	[1]	[2]	[3]
Value 1: It is important to love nature more	0.85	-	0.89
Value 2: It is important to stop environmental pollution	0.82	-	0.84
Value 3: It is important to protect and preserve environment	0.85	-	0.83
Value 4: It is important we shall live with nature	0.71	-	0.79
Value 5: It is important to help each other	-	0.69	0.71
Value 6: It is important to avoid war	0.69	-	0.67
Value 7: It is important to have equal justice	0.56	0.50	0.73
Value 8: It is important we shall have equal opportunity	-	0.80	-
Value 9: It is important we should take care of those who are worse off	-	0.81	-
Eigen value	4.90	1.10	4.30
Explained variance	3.65	2.33	-
Internal Consistency (Cronbach's Alpha)	0.89	0.79	0.89
Average importance	5.36	4.90	5.28

Note: [1] = *Biosphere*; [2] = *Altruism*; [3] = *Self-Transcendence*.

TABLE 3 | Construct reliability and validity.

Construct	Indicators	Factor loading	Cronbach's Alpha	AVE	CR	*p* (Rho)
**One-Factor:**	Value 1: It is important to love nature more	0.90***	0.890	0.558	0.896	0.909
1.Self-transcendence	Value 2: It is important to stop environmental pollution	0.84***	-	-	-	-
	Value 3: It is important to protect and preserve environment	0.82***	-	-	-	-
	Value 4: It is important we shall live with nature	0.76***	-	-	-	-
	Value 5: It is important to help each other	0.63***	-	-	-	-
	Value 6: It is important to avoid war	0.58***	-	-	-	-
	Value 7: It is important to have equal justice	0.64***	-	-	-	-
**Two-Factor:**						
1.Biosphere	Value 1: It is important to love nature more	0.92***	0.893	0.672	0.891	0.877
	Value 2: It is important to stop environmental pollution	0.81***	-	-	-	-
	Value 3: It is important to protect and preserve environment	0.80***	-	-	-	-
	Value 4: It is important we shall live with nature	0.74***	-	-	-	-
2.Altruism	Value 5: It is important to help each other	0.88***	0.786	0.561	0.832	0.945
	Value 7: It is important to have equal justice	0.86***	-	-	-	-
	Value 8: It is important we shall have equal opportunity	0.59***	-	-	-	-
	Value 9: It is important we should take care of those who are worse off	0.62***	-	-	-	-

Note: *** $p < 0.001$.

AVE: Average variance extracted; CR: composite reliability

TABLE 4 | Results for one-factor CFA model.

	Unstandardized value	Standardized value
Loadings (Self-transcendence)		
Value 1: It is important to love nature more	1.00 (fixed)	0.90***
Value 2: It is important to stop environmental pollution	0.91***	0.84***
Value 3: It is important to protect and preserve environment	0.78***	0.82***
Value 4: It is important we shall live with nature	0.89***	0.76***
Value 5: It is important to help each other	0.76***	0.63***
Value 6: It is important to avoid war	0.70***	0.58***
Value 7: It is important to have equal justice	0.81***	0.64***
Variances		
error. Value 1	0.12	0.20
error. Value 2	0.17	0.29
error. Value 3	0.15	0.33
error. Value 4	0.27	0.42
error. Value 5	0.42	0.60
error. Value 6	0.46	0.66
error. Value 7	0.45	0.59
Self-transcendence	0.49	1.00 (fixed)
Covariance		
error. Value 2 with error. Value 4	-0.05*	-0.22*

*** $p < 0.001$, ** $p < 0.01$, * $p < 0.05$.

TABLE 5 | Results of two-factor CFA model.

	Unstandardized value	Standardized value
Loadings (Biosphere)		
Value 1: It is important to love nature more	1.00 (fixed)	0.92***
Value 2: It is important to stop environmental pollution	0.86***	0.81***
Value 3: It is important to protect and preserve environment	0.85***	0.80***
Value 4: It is important we shall live with nature	0.74***	0.75***
Variances		
error. Value 1	0.09	0.15
error. Value 2	0.20	0.35
error. Value 3	0.16	0.36
error. Value 4	0.19	0.44
Biosphere	0.52	1.00 (fixed)
Covariance		
error. Value 2 with error. Value 3	0.03*	0.18*
Loadings (Altruism)		
Value 5: It is important to help each other	1.00 (fixed)	0.85***
Value 7: It is important to have equal justice	1.06***	0.85***
Value 8: It is important we shall have equal opportunity	0.89***	0.61***
Value 9: It is important we should take care of those who are worse off	0.77***	0.66***
Variances		
error. Value 5	0.20	0.28

error. Value 7	0.22	0.28
error. Value 8	0.66	0.63
error. Value 9	0.40	0.57
Altruism	0.50	1.00 (fixed)
Covariance		
error. Value 5 with error. Value 7	-0.17***	-0.82*
error. Value 7 with error. Value 9	-0.13**	-0.43*
Biosphere with altruism	0.37***	0.73***

*** $p < 0.001$, ** $p < 0.01$, * $p < 0.05$.

TABLE 6 | Effects of past volunteering in clean-up on pro-environmental knowledge, intention, and practices.

	Pro-Environmental knowledge, attitude, and practices							
	[1]	[2]	[3]	[4]	[5]	[6]	[7]	[8]
Volunteering	-0.077	-0.101	-0.068	0.012	-0.243	0.034	0.045	0.039
	(0.172)	(0.130)	(0.161)	(0.221)	(0.163)	(0.105)	(0.134)	(0.154)
Male	-0.274^{+}	-0.135	0.277^{+}	-0.153	0.038	0.004	-0.506***	0.157
	(0.160)	(0. 124)	(0.157)	(0.195)	(0.153)	(0.101)	(0.132)	(0.141)
Age	-0.024^{+}	-0.009	0.022^{+}	-0.006	0.001	0.004	-0.012	-0.007
	(0.014)	(0.011)	(0.013)	(0.016)	(0.013)	(0.009)	(0.010)	(0.012)
Education	0.022	-0.020	-0.140**	0.015	-0.030	0.015	0.009	0.024
	(0.050)	(0.043)	(0.053)	(0.060)	(0.043)	(0.030)	(0.047)	(0.042)
Self-transcendence	-0.335**	-0.341***	-0.058	-0.334*	-0.108	0.220*	0.254*	0.035
	(0.118)	(0.088)	(0.102)	(0.145)	(0.114)	(0.098)	(0.102)	(0.113)
Fixed effect (home-country)	-0.188	-0.385*	0.370^{+}	0.589*	-0.319^{+}	-0.142	0.550**	-0.046
	(0.200)	(0.165)	(0.203)	(0.258)	(0.183)	(0.135)	(0.177)	(0.166)
Fixed effect (host-country)	-0.278	-0.342*	0.111	1.037***	-0.029	0.047	0.877***	-0.179
	(0.203)	(0.163)	(0.193)	(0.239)	(0.181)	(0.132)	(0.181)	(0.179)
Constants	5.238***	5.099***	5.179***	4.511***	4.468***	1.822*	1.240	3.015**
	(0.994)	(0.791)	(0.950)	(1.215)	(0.877)	(0.734)	(0.896)	(0.858)
Observations (*N*)	210	210	209	209	209	209	208	209
R-squared (R^2)	0.074	0.112	0.075	0.098	0.046	0.051	0.225	0.010

Note: Multiple Linear Regression (OLS) is used. [1]: Do you agree waste is difficult to separate? [2]: Do you agree you don't have time to separate your garbage? [3]: Do you agree waste separate need the involvement of your neighbors? [4]: Do you agree waste separation need the improved collection system? [5]: Do you agree waste separation need the involvement of family members? [6]: Do you intend to separate your garbage? [7]: How often do you use shopping-bag? [8]: Do you agree waste should be burned or buried?
VIFs: collinearity and multicollinearity, the problems of combined variables. Mean of Variance Inflation Factors (VIFs) = 1.36
Standard errors are in parentheses.
*** $p < 0.001$, ** $p < 0.01$, * $p < 0.05$.

FIGURE 1 | Indices of one-factor and two-factor CFA Model.

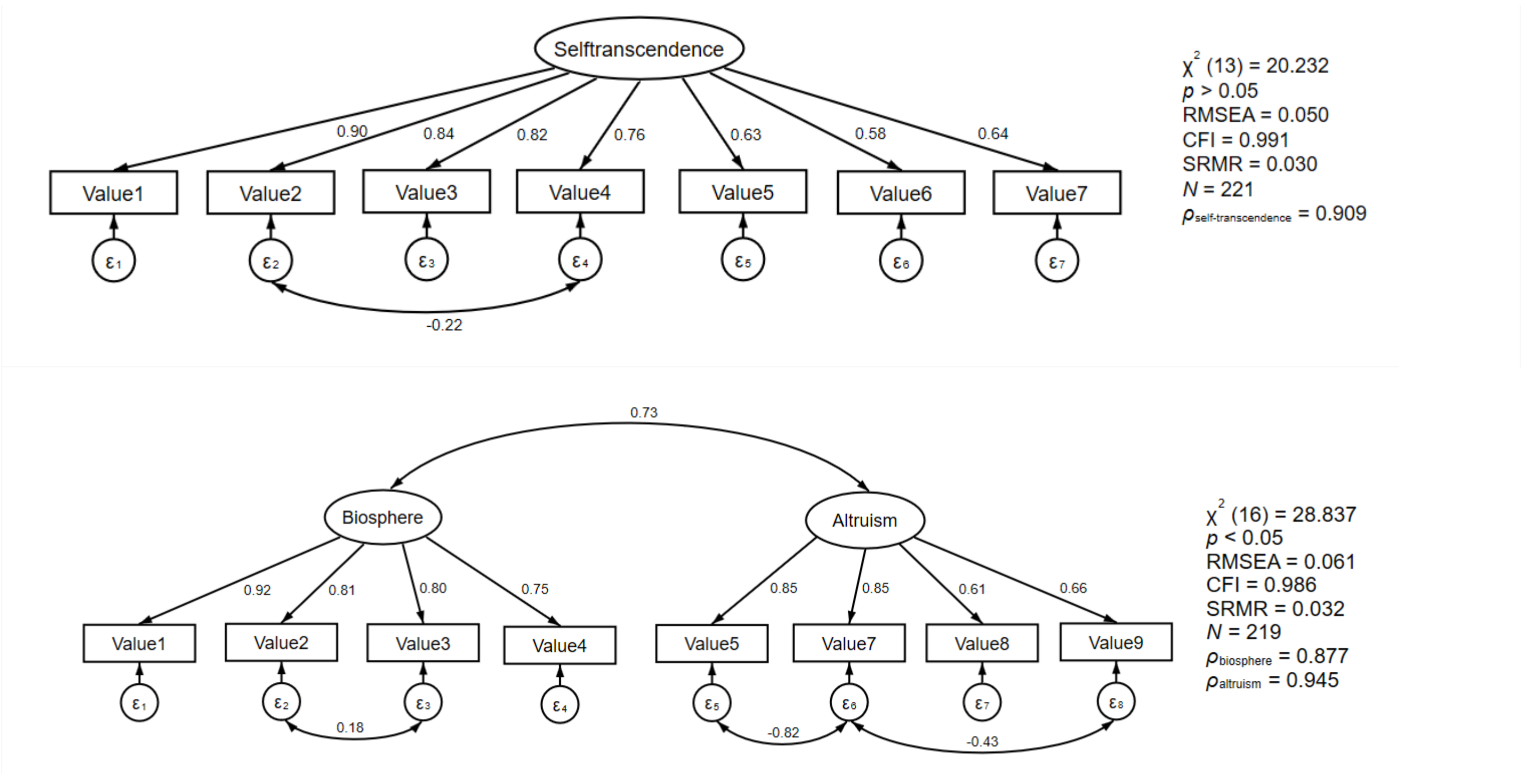

Supplement: Supplementary file 1 [file Data_Sheet_1.PDF]
